# Assessment of malignant potential for HPV types 16, 52, and 58 in the uterine cervix within a Korean cohort

**DOI:** 10.1038/s41598-024-65056-7

**Published:** 2024-06-25

**Authors:** Juhun Lee, Dong Ja Kim, Hyun Jung Lee

**Affiliations:** 1 Department of Obstetrics and Gynecology, School of Medicine, Kyungpook National University, Kyungpook National University Hospital, 130, Dongdeok-ro, Jung-gu, Daegu, 41944 Republic of Korea; 2 Department of Forensic Medicine, School of Medicine, Kyungpook National University, Kyungpook National University Hospital, Daegu, Republic of Korea

**Keywords:** human papillomavirus, cervical pre-malignancy, malignant potential, risk of carcinogenesis, cervical cancer, Cancer, Infectious diseases, Urogenital diseases, Risk factors

## Abstract

High-risk human papillomavirus (HR-HPV) is the primary carcinogen in uterine cervical carcinoma. While genotype-specific carcinogenic risks have been extensively studied in Western populations, data from Korean are sparse. This study evaluates the malignant potential of the three most prevalent HR-HPVs in Korea: HPV16, HPV52, and HPV58. We analyzed 230 patients who underwent cervical conization and had been tested for HPV within a year prior to the procedure, excluding those with multiple infections. This analysis was confined to patients with single HPV infections and assessed outcomes of CIN3+, which includes carcinoma in situ (CIN3) and invasive carcinoma. The incidence of invasive cervical cancer was 6.7% for HPV16, 1.7% for HPV52, and 2.0% for HPV58; however, these differences were not statistically significant (*p* = 0.187). The rate of CIN3+ for HPV16, HPV52, and HPV58 were 70.6%, 51.7%, and 58.8%, respectively. Despite the small sample size, which may limit the robustness of statistical analysis, the data suggest a higher observed risk with HPV16. These findings highlight the need for vigilant clinical management tailored to specific HPV genotypes and support the implementation of a nine-valent vaccine in Korea. Physicians should be aware of these genotype-specific risks when treating patients.

## Introduction

Uterine cervical cancer including squamous cell carcinoma and adenocarcinoma is the fourth most common cancer worldwide^1^ and the fifth one in women in South Korea^2^. High-risk human papillomavirus (HR-HPV) is a critical carcinogen in uterine cervical carcinoma, and persistent infection with this virus accounts for 99.7% of all cervical cancer incidences^3–5^. More than 200 distinct HPV-types have been identified^6–8^. In 1995, the International Agency for Research on Cancer classified HPV16 and HPV18 as cervical carcinogens. This list was expanded in 2011 to include twelve high-risk HPV types: HPV31, HPV33, HPV35, HPV39, HPV45, HPV51, HPV52, HPV56, HPV58, and HPV59^9,10^. HR-HPVs have been reported to have different distributions according to geographical regions or countries; however, in general, HPV16 and HPV18 are the two most prevalent worldwide8-16. However, whether the malignant potential of each HR-HPV is similar or significantly different remains unclear in Asia whereas well documented in the Western world^11–13^.

HPV infects the basal layer of the squamous epithelium and causes malignant progression with alterations of various signaling pathways in the uterine cervix^14,15^. Among HPV oncogenes, E6 and E7 play a significant role in HPV-induced carcinogenesis^16^. The E6 viral protein binds to p53 and inhibits its function via ubiquitin-dependent degradation, whereas the E7 protein promotes cell proliferation via pRB inhibition^17,18^. Genomic instability and tumor-promoting host cell mutations are induced by the function of these proteins and their interactions with other signaling molecules^14^.

Two previous studies conducted in Korea have assessed the malignant potential of single infections with HR-HPV. However, these studies have some limitations due to unclear or poorly defined exclusion criteria^19,20^.

This study aimed to evaluate the malignant potential of the three most prevalent HR-HPV genotypes in Korea with thorough control of clinical factors.

## Methods

### Patients

**Figure 1:**
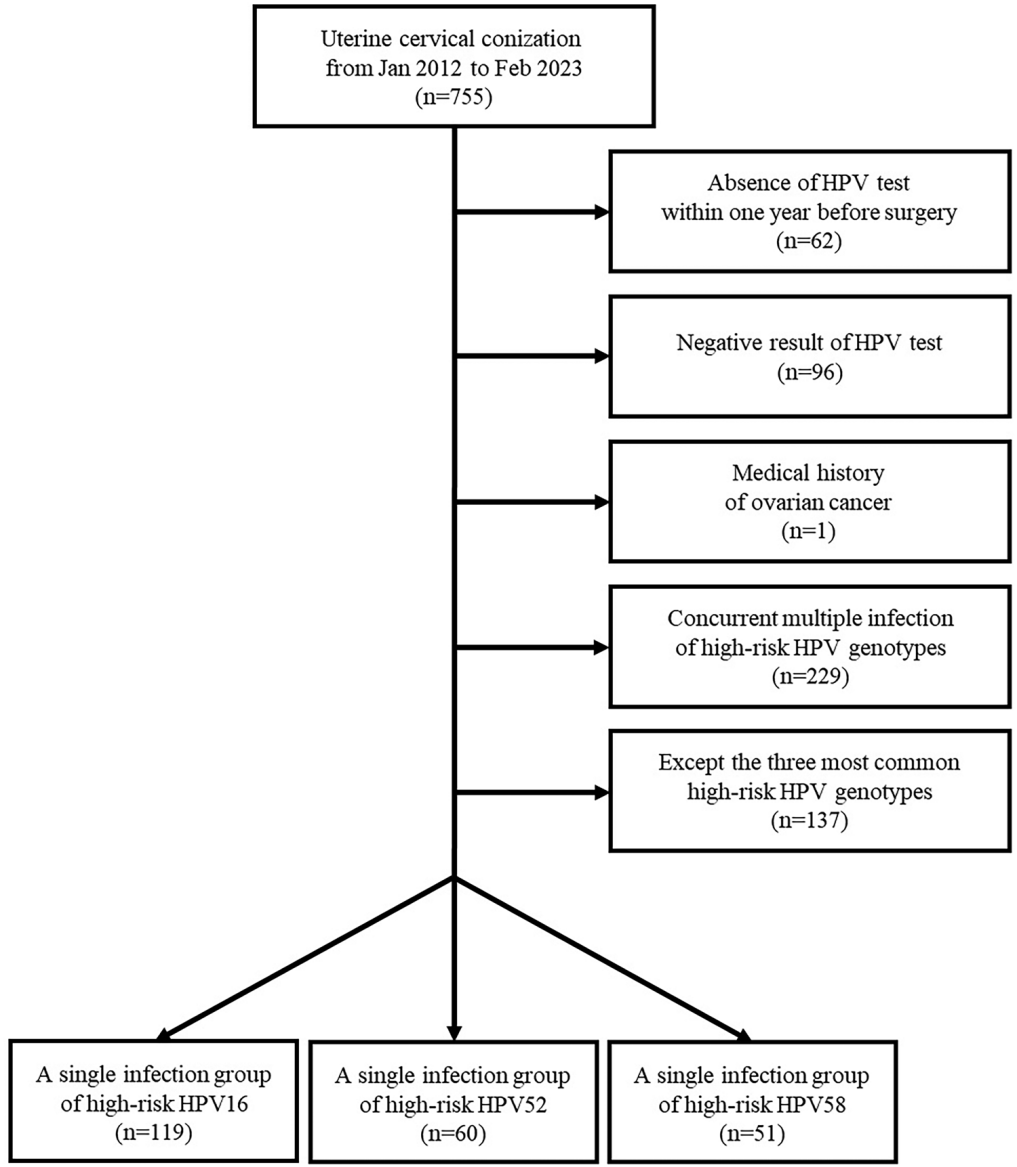
Flow diagram for patient selection.

We retrospectively reviewed consecutive 755 patients who underwent uterine cervical conization for the diagnosis or treatment of cervical pre-malignant lesions or carcinoma from January 2012 to February 2023 in Kyungpook National University Hospital (KNUH). Sixty-two patients were excluded owing to the absence of HPV test within 1 year preoperatively. Additionally, 96 patients were excluded owing to the negative HR-HPV result on the HPV test. One woman was excluded because of her medical history of ovarian cancer. We excluded 229 patients because of concurrent multiple HR-HPV infections, two or more HR-HPV genotypes, on the test. Of the patients who appeared to exhibit a single HR-HPV infection, we included the three most prevalent HR-HPV genotypes. The flow diagram for patient selection is presented in Fig. 1. Informed consent was waived by the Institutional Review Board of Kyungpook National University Hospital because of the retrospective nature of the study. All methods in this study were in accordance with the Declaration of Helsinki. This study was approved by the Institutional Review Board of Kyungpook National University Hospital (KNUH 2023-06-015).

### Definition of initial and final diagnoses

The initial diagnosis was determined on the basis of the pathologic result of cervical swab cytology, the Pap smear or liquid-based thinPrep, along with the 2001 Bethesda System or cervical biopsy^21^. Inflammation or reactive change, or benign cervical polyp was classified as normal. The atypical cells included atypical squamous cells (ASCs), ASC of undetermined significance, ASC cannot exclude high-grade lesion, atypical glandular cells (AGC), and AGCs of undetermined significance. The final diagnosis was determined based on the pathologic result from uterine cervical conization. Acute or chronic inflammation was classified as normal. In the initial or final diagnosis, low-grade cervical intraepithelial neoplasia (LSIL or CIN1) included cases of koilocytosis without definite dysplasia or dysplasia where the grade could not be determined. High-grade cervical intraepithelial neoplasia (HSIL) encompassed both CIN2 and CIN3; CIN3 also included cases previously classified under carcinoma in situ (CIS) in other systems. In initial diagnoses where invasiveness was unclear, both CIN3 and invasive cervical cancer were considered. When lesions of two different grades, such as CIN1 and CIN2/CIN3, were detected simultaneously, the diagnosis reflected the grade with the higher malignant potential. An experienced gynecologic pathologist (D.J. Kim) reviewed and validated the final diagnosis.

### Statistical analysis

Categorical variables were evaluated using the Chi-square test or Fisher’s exact test, whereas continuous variables were compared using one-way analysis of variance and the Scheffe test for *post-hoc* analysis. Logistic regression was used to analyze the risk of invasive cervical cancer among the three most prevalent HR-HPV genotypes. A *p*-value of < 0.05 was considered statistically significant. All statistical analyses were performed using Statistical Package for the Social Sciences version 26 (IBM Corp., Armonk, NY, USA).

### Uterine cervical conization

The loop electrosurgical excision procedure was adopted to perform uterine cervical conization. In this study, a total of 15 surgeons performed this surgery. According to the decision of the surgeon, endocervical conization and/or endocervical curettage were also performed. To determine the resection margin, colposcopy using acetic acid was employed.

### HPV test

The sample for the HPV test was harvested using a uterine cervical swab. This sample was sent to a pathologist for the test via a liquid medium. To determine the HPV infection status, two different methods were employed, including DNA microarray and real-time polymerase chain reaction (RT-PCR), owing to their similar validation^22,23^. The HPV tests were not centrally reviewed, and the titer of HPV DNA was not taken into consideration. Thus, the results in local medical institutions, not only in our institution, were also included in this study.

## Results

Of the 230 included patients, 119 (51.7%) tested positive for HPV16, 60 (26.1%) for HPV52, and 51 (22.8%) for HPV58, respectively. Among the women studied, no significant difference was observed in the distribution between the initial diagnosis, such as normal, atypical cells, LSIL, HSIL, invasive carcinoma, based on cervical biopsy or cervical swab cytology (*p* = 0.540, *p* = 0.250, *p* = 0.390, *p* = 0.261, *p* = 0.569, respectively) (Table 1).

**Table 1:** Characteristics and clinical factors of patients with a single HR-HPV genotype infection.

	Single infection group with HPV 16	Single infection group with HPV 52	Single infection group with HPV 58	*p*-value*
	(n = 119)	(n = 60)	(n = 51)	
Age (years)	45.38 ± 12.87	48.42 ± 12.46	45.94 ± 12.89	0.317
Initial diagnosis (n)				
Normal	1 (0.8%)	0 (0%)	1 (2.0%)	0.540
Atypical cells	9 (7.6%)	9 (15.0%)	4 (7.8%)	0.250
LSIL^†^	10 (8.4%)	9 (15.0%)	5 (9.8%)	0.390
HSIL^‡^	94 (79.0%)	41 (68.3%)	40 (78.4%)	0.261
Invasive carcinoma^§^	5 (4.2%)	1 (1.7%)	1 (2.0%)	0.569

The initial diagnosis is determined on the basis of cervical swab cytology or punch biopsy.

Data are expressed as numbers (%) and means ± standard deviations.

LSIL, low-grade squamous intraepithelial lesion; HSIL, high-grade squamous intraepithelial lesion.

*Statistical significance is evaluated using one-way analysis of variance with *post-hoc* analysis in the age and Chi-square test in the initial diagnosis.

^†^Included koilocytosis without definite dysplasia or dysplasia for which the grade cannot be determined.

^‡^Included carcinoma in situ.

^§^Regardless of histologic subtypes, such as squamous cell carcinoma or adenocarcinoma.

In the final diagnoses, no significant differences were found in the distribution, except CIN2. HPV16 exhibited significantly less CIN2 compared to HPV52 (2 [1.7%] vs. 11 [18.3%], *p* < 0.001) or HPV58 (2 [1.7%] vs. 6 [11.8%], *p* < 0.010). However, there were no significant differences in the distribution of CIN3 between HPV16, HPV52, and HPV58 (76 [63.9%] vs. 30 [50.0%] vs. 29 [56.9%], respectively, *p* = 0.197). Invasive cervical cancer was diagnosed in 10 patients: 8 with HPV16, 1 with HPV52, and 1 with HPV58. The corresponding risks of developing invasive cervical cancer were 6.7% for HPV16, 1.7% for HPV52, and 2.0% for HPV58 (*p* = 0.187). The CIN3+ ratio was significantly higher in HPV16 compared to HPV52 (84 [70.6%] vs. 31 [51.7%], (*p* = 0.014), but not significantly higher than HPV58 (84 [70.6%] vs. 30 [58.8%], *p* = 0.156) (Table 2).

**Table 2:** Comparison of final diagnoses among the high-risk HPV16, HPV52, and HPV58 single infection groups.

	Whole group				Subgroup (≤ 50 years)				Subgroup (> 50 years)			
	HPV16	HPV52	HPV58	*p*-value*	HPV16	HPV52	HPV58	*p*-value	HPV16	HPV52	HPV58	*p*-value
	(n = 119)	(n = 60)	(n = 51)		(n = 79)	(n = 38)	(n = 32)		(n = 40)	(n = 22)	(n = 19)	
Normal	19 (16.0%)	10 (16.7%)	6 (11.8%)	0.733	13 (16.5%)	4 (10.5%)	4 (12.5%)	0.660	6 (15.0%)	6 (27.2%)	2 (10.5%)	0.319
CIN1	14 (11.8%)	8 (13.3%)	9 (17.6%)	0.588	5 (6.3%)	6 (15.8%)	5 (15.6%)	0.182	9 (22.5%)	2 (9.1%)	4 (21.1%)	0.407
CIN2	2 (1.7%)	11 (18.3%)	6 (11.8%)	< 0.001	2 (2.5%)	9 (23.7%)	6 (18.8%)	0.001	0 (0%)	2 (9.1%)	0 (0%)	0.064
CIN3^†^	76 (63.9%)	30 (50.0%)	29 (56.9%)	0.197	56 (70.9%)	19 (50.0%)	17 (53.1%)	0.049	20 (50.0%)	11 (50.0%)	7 (36.8%)	0.603
Invasive carcinoma^‡^	8 (6.7%)	1 (1.7%)	1 (2.0%)	0.187	3 (3.8%)	0 (0%)	0 (0%)	0.258	5 (12.5%)	1 (4.5%)	1 (5.3%)	0.473
CIN3 + ^§^	84 (70.6%)	31 (51.7%)	30 (58.8%)	0.036	59 (74.7%)	19 (50.0%)	17 (53.1%)	0.013	25 (62.5%)	12 (54.5%)	13 (68.4%)	0.654

Final diagnosis is determined on the basis of the pathologic examination following uterine cervical conization.

Data are expressed as numbers (%).

CIN, Cervical intraepithelial neoplasm.

*All statistical significances are evaluated using the Chi-square test.

^†^Includes carcinoma in situ or adenocarcinoma in situ, either.

^‡^Regardless of histologic subtypes, such as squamous cell carcinoma or adenocarcinoma.

^§^Includes CIN3 and invasive carcinoma.

Logistic regression analysis was conducted to evaluate the risk of malignant potential in HPV16 vs. HPV52, HPV16 vs. HPV58, and HPV52 vs. HPV58. For CIN3+, HPV16 showed significantly higher risk than HPV52 (odds ratio [OR] = 2.245, 95% confidence interval [CI] = 1.182–4.265, *p* = 0.014). Between HPV16 and HPV58, the OR for CIN3+ was 1.680 (95% CI = 0.849–3.326, *p* = 0.137). Between HPV52 and HPV58, the OR for CIN3+ was 0.748 (95% CI = 0.352–1.589, *p* = 0.451). For invasive carcinoma, there were no significant ORs. Between the HPV16 and HPV52, the OR was 4.252 (95% CI = 0.519–34.819, *p* = 0.177). Between the HPV16 and HPV58, the OR was 3.604 (95% CI = 0.439–29.590, *p* = 0.233). Between the HPV52 and HPV58, the OR was 0.847 (95% CI = 0.052–13.898, *p* = 0.908).

## Discussion

The three most prevalent HR-HPV types among screened women in Korea are HPV16, HPV52, and HPV58. In our study, the cases of invasive cervical cancer associated with these types were 8 for HPV16, and 1 each for HPV52 and HPV58, corresponding to cancer risks of 6.7% for HPV16, 1.7% for HPV52, and 2.0% for HPV58, respectively. Despite HPV16 being the most prevalent, the data show a higher relative risk of invasive cancer with this genotype compared to HPV52 and HPV58. Additionally, the risk of requiring medical intervention was higher in HPV16 single infection group compared to the HPV52 or HPV58 single infection group; significantly higher than HPV52 and not significant compared to HPV58.

The results of this study can assist physicians explaining the malignant potential of HR-HPV genotypes 16, 52, and 58 to their patients. Physicians should emphasize that the malignant potential of HPV52 or HPV58 can have similar to that of HPV16. Furthermore, since the quadrivalent vaccine does not cover HPV52 or HPV58, our findings support the necessity for a nine-valent vaccine against HR-HPVs. Such a vaccine would cover HPV6, HPV11, HPV16, HPV18, HPV31, HPV33, HPV45, HPV52, and HPV58.

To evaluate the malignant potential while controlling for age, subgroup analysis was performed. The entire patient cohort was divided into two subgroups on the basis of age, one with individuals aged 50 years or younger and the other with individuals older than 50 years. In the subgroup of individuals aged 50 years or younger, the number of HPV16, HPV52, and HPV58 single infection groups were 79 (53.0%), 38 (25.5%), and 32 (21.5%) cases, respectively. The prevalence of CIN3+ was observed in 59 (74.7%), 19 (50.0%), and 17 (53.1%) cases, respectively. In this subgroup, the CIN3+ ratio was significantly higher in HPV16 compared to HPV52 (*p* = 0.008) or HPV58 (*p* = 0.041) whereas the ratio of CIN2 in HPV16 was significantly lower compared to HPV52 (*p* = 0.001) or HPV58 (*p* = 0.007). Logistic regression showed that HPV16 had a significantly higher risk for CIN3+ compared to HPV52 (OR = 2.950, 95% CI = 1.308–6.654, *p* = 0.009) or HPV58 (OR = 2.603, 95% CI = 1.102–6.150, *p* = 0.029) whereas not significant in HPV52 vs. HPV58 (OR = 0.882, 95% CI = 0.344–2.262, *p* = 0.794). In the subgroup of individuals older than 50 years, 40 (49.4%), 22 (27.2%), and 19 (23.5%) cases of HPV16, HPV52, and HPV58, respectively, were noted. The prevalence of CIN3+ was observed in 25 (62.5%), 12 (54.5%), and 13 (68.4%) cases, respectively. Unlike with the subgroup of individuals aged 50 years or younger, no significant differences were noted in the CIN3+ ratio, and the risk of CIN3+ was not significantly different in the logistic regression test (HPV16 vs. HPV52, OR = 1.389 [95% CI = 0.483–3.991, *p* = 0.542]; HPV16 vs. HPV58, OR = 0.769 [95% CI = 0.241–2.454, *p* = 0.658]; HPV52 vs. HPV58, OR = 0.554 [95% CI = 0.154–1.993, *p* = 0.366]) (Table 2).

In Tables 1 and 2, we identified a discrepancy between the ratios of HSIL in the initial diagnosis and the sum of CIN2 and CIN3 in the final diagnosis within all three single infection groups. As mentioned in the Methods section, the initial diagnosis was determined on the basis of cervical swab cytology or punch biopsy in the office. Therefore, we assessed the diagnostic accuracy of cervical swab cytology and punch biopsy under colposcopy with acetic acid application to detect high grade lesions, such as CIN2, CIN3, or invasive carcinoma. All patients, regardless of their HPV infection status or whether they had undergone an HPV test within 1 year before conization, were included in this analysis, except for patients without cervical swab cytology or biopsy under colposcopy results. The pathologic diagnoses of both screening tests and cervical conization were categorized into CIN2− and CIN2+, with CIN2− encompassing normal, atypical cells, and CIN1, whereas CIN2+ included CIN2, CIN3, and invasive carcinoma.

In the cervical swab cytology for detecting CIN2+ (n = 738), the sensitivity (SS), specificity (SP), positive predictive value (PPV), and negative predictive value (NPV) was 32.8% (150/458), 86.1% (241/280), 79.4% (150/189), and 43.9% (241/549), respectively. The overall diagnostic accuracy was 53.0% (391/738). In the cervical punch biopsy under colposcopy with acetic acid application for detecting CIN2+ (n = 597), the SS, SP, PPV, and NPV was 89.5% (365/408), 42.3% (80/189), 77.0% (365/474), and 65.0% (80/123), respectively. The overall diagnostic accuracy was 74.5% (445/597).

Based on some recent studies, the Pap smear appeared to have a sensitivity of 47.2–55.5% and a specificity of 64.8–75.0%, whereas cervical biopsy under colposcopy showed a sensitivity and specificity of 64.7% and 52.74%, respectively^24^. Considering our data, cervical swab cytology and colposcopy examination appear to offer the benefits of high specificity and high sensitivity, respectively. However, as these examinations can exhibit low reproducibility, further studies are needed to establish optimal guidelines, especially in limited or resource-constrained condition.

**Figure 2:**
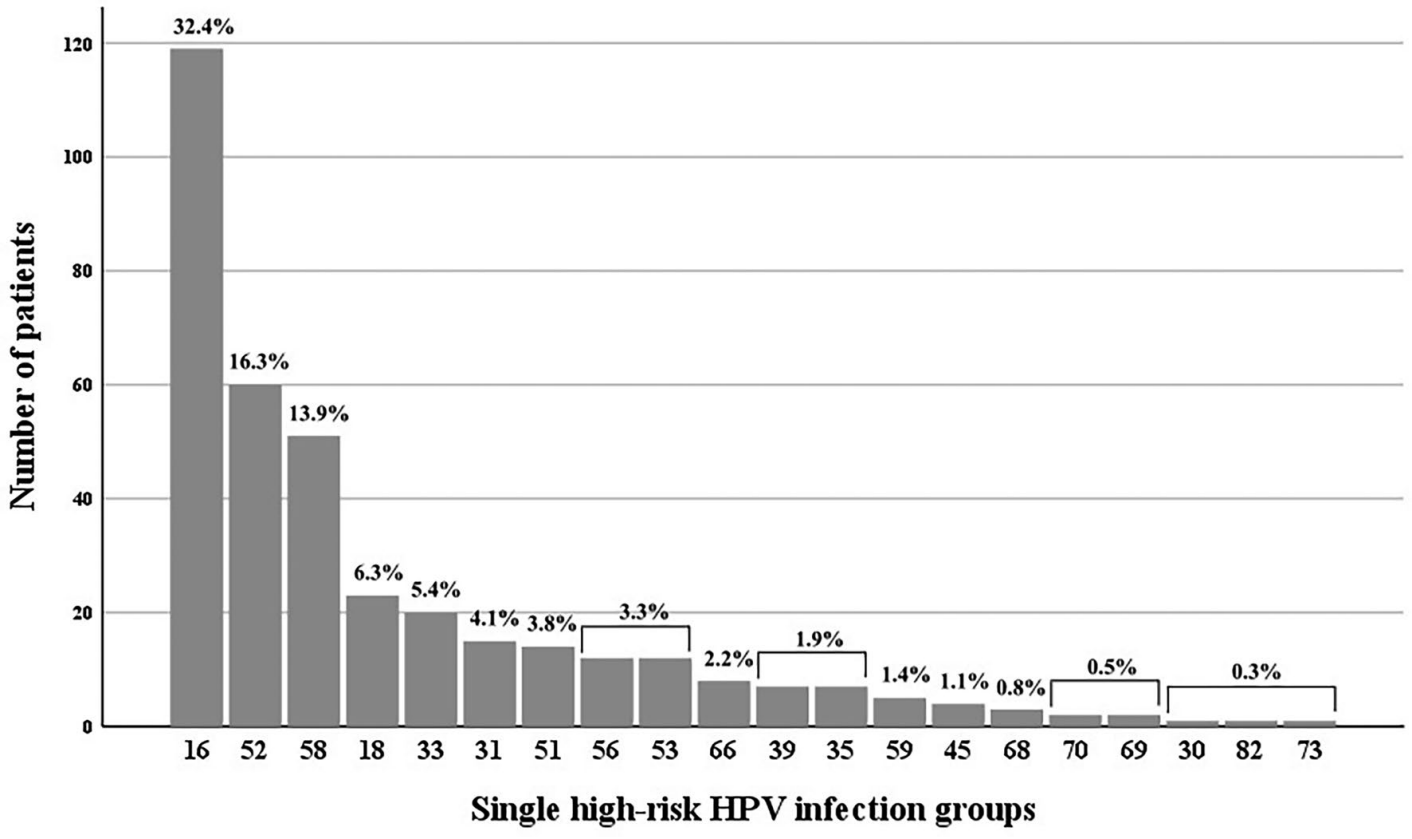
Graph presenting the count and ratio of each high-risk HPV genotype, including all genotypes not only HPV16, HPV52, and HPV58, among the patients enrolled in this study. All subgroups represent the single infection group.

The count and ratio of each HR-HPV genotype including all genotypes not only HPV16, HPV52, and HPV58 among the patients enrolled in this study are presented in Fig. 2. All subgroups represent the single infection group. The 10 most prevalent genotypes, in descending order, were HPV16, HPV52, HPV58, HPV18, HPV33, HPV31, HPV51, HPV53, HPV56, and HPV66. Among these, excluding HPV16, HPV52, and HPV58 which have already been mentioned above, the HPV genotypes with more than 10 patients were HPV18, HPV31, HPV33, HPV51, HPV53, and HPV56 (23, 15, 19, 14, 12, and 12 patients respectively). The rates of CIN3+ for these HR-HPV genotypes were 47.8%, 40.0%, 73.7%, 35.7%, 25.0%, and 25.0% respectively (11, 6, 14, 5, 3, and 3 patients respectively).

Recent studies have analyzed the HPV genotype-specific risk for carcinogenesis in the uterine cervix^19,20^. Park E et al. evaluated the risk on the basis of cervical biopsy. In this study, we identified HPV16, HPV52, and HPV58 as the three most common HR-HPV genotypes in Korean women, which is consistent with the reports of those authors. However, unlike the results of their study, our results were based on the pathologic results of uterine cervical conization. Moreover, they did not elucidate whether the patients infected with multiple HR-HPV genotypes were excluded. Thus, although they demonstrated a significantly higher carcinogenic risk for several HR-HPV genotypes including HPV16, HPV52, and HPV58 and a much higher OR of HPV16 than other genotypes, multiple infections with HR-HPV genotypes may have influenced these results. In this study, the HPV 16-, HPV52-, and HPV58-specific risk for carcinogenesis was clearly shown owing to the exclusion of concurrent multiple HR-HPV infections. On the basis of age, the authors stratified the patients into the following five subgroups: ≤ 34, 35–44, 45–54, 55–64, and ≥ 65 years. This enabled them to evaluate age- and HR-HPV genotype-specific risks^20^.

Another similar study conducted by So KA et al. was based on cervical biopsy results. They did not describe the inclusion or exclusion criteria for age; based on the result, the authors seemed to have included patients of all ages. In their study, HR-HPV genotypes 16, 52, and 58 were the most prevalent, which is consistent with our results. Their results showed that some HR-HPV genotypes such as 16, 31, 33, 52, and 58 were significantly more in CIN2, CIN3, and cervical cancer than those in the normal or CIN1 group. However, as patients with concurrent multiple infections were included in their study, the genotype-specific risk was unclear^19^.

The strength of this study was the homogeneous cohort with a single infection of HPV16, HPV52, or HPV58. This enabled the study to evaluate the malignant potential of each genotype with less bias compared with previous studies.

This study had some limitations. First, this was a retrospective study with a small sample size from a single center. Owing to the small sample size, the cohort could not be further stratified on the basis of age. Therefore, bias may arise from the different sexual activities according to age. A selection bias might also have affected the results because some patients, postmenopausal or not wanting more pregnancies, could have chosen to receive hysterectomy due to high grade lesion, such as CIN2, CIN3, or microinvasive carcinoma rather than cervical conization. Second, several patients in the cohort did not undergo follow-up HPV test before surgery. Thus, we could not prove or control the influence of persistent HR-HPV infection despite it being known as a critical factor in cervical cancer^4,5^. Third, we could not review the HPV vaccination history. The Korean government included the quadrivalent and bivalent HPV vaccines (against HPV6, HPV11, HPV16, and HPV18; against HPV16 and HPV18) in the National Program 2016 and provided these vaccines for girls aged 12–17 years. According to statistics from the Korean government, approximately 70% of Korean girls have received the vaccines^25^. Finally, the HPV test in this study included two different methods, including DNA microarray and RT-PCR. Heterogeneity might have arisen from this aspect, raising concerns about the reliability of detecting HPV genotypes. When the titration of a certain HPV DNA was very high, other HPV genotypes’ DNA could not have been detected on the HPV test. Some patients could have had concurrent multiple infection of HR-HPVs, even though they had been shown to have a single HR-HPV infection on the HPV test. Furthermore, the HPV test was not centrally reviewed.

## Conclusion

Our investigation into the malignant potential of the three most prevalent HR-HPV types in the Korean population–HPV16, HPV52, and HPV58–indicates that while HPV16 showed a higher incidence of invasive cervical cancer (6.7%) compared to HPV52 (1.7%) and HPV58 (2.0%), these differences were not statistically significant (*p* = 0.187). This suggests that while HPV16 is more prevalent, the risk of developing invasive cervical cancer from any of these types is similarly concerning within the observed cohort.

The data reinforce the necessity of including HPV52 and HPV58 in clinical screening and vaccination strategies, alongside HPV16, to comprehensively address the risk of cervical cancer. The findings advocate for the adoption of broader preventive measures, such as the inclusion of these HPV types in vaccination programs in Korea, reflecting the global shift towards vaccines that cover multiple HR-HPV types.

Despite the lack of statistically significant differences among the HPV types in terms of progressing to invasive cancer, our results highlight the importance of continued vigilance in screening for all HR-HPV infections. The consistent risk across these types supports the universal application of preventive strategies, including vaccination and regular cervical screening to mitigate the overall burden of cervical cancer.

This study underlines the need for further research into the oncogenic potential of various HPV types within different populations to tailor public health initiatives effectively and ensure that prevention strategies are based on robust epidemiological evidence.

## Data Availability

The data that support the findings of this study are available on reasonable request from the corresponding author.
